# Differential immunological profiles in seronegative versus seropositive rheumatoid arthritis: Th17/Treg dysregulation and IL-4

**DOI:** 10.3389/fimmu.2024.1447213

**Published:** 2024-09-03

**Authors:** Baochen Li, Rui Su, Qiaoling Guo, Ronghui Su, Chong Gao, Xiaofeng Li, Caihong Wang

**Affiliations:** ^1^ Department of Rheumatology, Second Hospital of Shanxi Medical University, Taiyuan, Shanxi, China; ^2^ Shanxi Key Laboratory of Immunomicroecology, Taiyuan, Shanxi, China; ^3^ Department of Geriatrics, Heping Hospital Affiliated to Changzhi Medical College, Changzhi, Shanxi, China; ^4^ Department of Pathology, Brigham and Women’s Hospital, Harvard Medical School, Boston, MA, United States

**Keywords:** seronegative rheumatoid arthritis, regulatory T cells, T-helper 17 cells, interleukin-4, biomarker

## Abstract

**Background:**

Rheumatoid arthritis (RA) is an autoimmune disease with various subtypes. Among these, seronegative rheumatoid arthritis (SnRA), distinguished by its distinctive seronegative antibody phenotype, presents clinical diagnosis and treatment challenges. This study aims to juxtapose the immunological features of SnRA with seropositive rheumatoid arthritis (SpRA) to investigate potential mechanisms contributing to differences in antibody production.

**Methods:**

This study included 120 patients diagnosed with RA and 78 patients diagnosed with psoriatic arthritis (PsA), comprising 41 cases of SnRA and 79 cases of SpRA. Clinical, serological, and immune data were collected from all participants to systematically identify and confirm the most pivotal immunological distinctions between SnRA and SpRA.

**Results:**

(1) SpRA demonstrates more pronounced T-helper 17 cells (Th17)/Regulatory T cells (Treg) dysregulation, vital immunological differences from SnRA. (2) SpRA exhibits higher inflammatory cytokine levels than SnRA and PsA. (3) Lymphocyte subset ratios and cytokine overall distribution in SnRA close to PsA. (4) Interleukin-4 (IL-4) emerges as the central immunological disparity marker between SnRA and SpRA.

**Conclusion:**

Th17/Treg imbalance is one of the vital immunological disparities between SnRA and SpRA. Interestingly, PsA and SnRA display similar peripheral blood immunological profiles, providing immunological evidence for these two diseases’ clinical and pathological similarities. Furthermore, IL-4 emerges as the central immunological disparity marker between SnRA and SpRA, suggesting its potential role as a triggering mechanism for differential antibody production.

## Background

Rheumatoid arthritis (RA) is a systemic autoimmune disease characterized by persistent synovial inflammation, leading to cartilage damage and bone invasion (1). As research advances, RA is increasingly recognized as a clinical syndrome encompassing different disease subtypes, treatment responses, and disease outcomes (2). The etiology of RA remains incompletely understood, although autoantibodies such as rheumatoid factor (RF) and anti-citrullinated protein antibodies (ACPAs) are used as biomarkers for RA diagnosis (3, 4). Particularly, ACPA detection has become internationally acknowledged for early RA diagnosis and assessing joint damage, being a specific and sensitive indicator (5). While most RA patients exhibit ACPAs and RF, approximately 25% of RA patients test negative for both ACPA and RF. Additionally, some patients are positive for only RF or ACPAs (6). Clinically, RA is often classified based on the presence or absence of RF and ACPAs into seropositive rheumatoid arthritis (SpRA) and seronegative rheumatoid arthritis (SnRA) (5). Previous research suggests that the coexistence of RF and ACPAs in RA patients may lead to more severe inflammation and higher disease activity (7). While significant advancements have been made in the treatment of SpRA over the past two decades, the clinical prognosis of SnRA patients, characterized by their unique antibody phenotype, has not progressed accordingly (8). This is particularly challenging as SnRA patients exhibit antibody phenotypes and clinical symptoms similar to those with Psoriasis Arthritis (PsA) (9). Therefore, elucidating the immunological factors underlying the differential expression of autoantibodies in SpRA and SnRA is crucial for targeted therapy of different RA subtypes.

T and B lymphocyte imbalance and inflammatory cytokine production are pivotal immunological factors in RA pathogenesis (10). However, there’s limited research on lymphocyte subsets and cytokine profiles in SnRA and SpRA patients. This study aims to analyze these differences and identify core immunological disparities using logistic regression and random forest analysis. Additionally, including PsA patients will help explore immunological similarities between SnRA and PsA, shedding light on potential triggers for differential antibody production in SnRA and SpRA.

## Methods

### Clinical data collection

This study included 41 patients with SnRA, 79 with SpRA, and 78 with PsA. Of these, there were 69 male patients and 129 female patients. We also included an cohort of 53 healthy individuals. The mean age of this control group was 47.68 ± 11.80 years, with males comprising 32% of the population. All RA patients were diagnosed according to the revised 1987 (11)and 2010 (12)ACR/EULAR classification criteria for RA, while PsA patients were diagnosed according to the revised 2006 CASPAR classification criteria (13). Patients with other autoimmune diseases, severe infections, tumors, and pregnant women were excluded. RA patients were classified as SnRA if both RF IgM and ACPA antibodies were negative at any time during the disease course and as SpRA if RF and/or ACPA antibodies were positive. Clinical data and laboratory parameters were retrospectively collected. Flow cytometry was used to detect the number and proportion of peripheral lymphocyte subsets, including total T cells, total B cells, CD4+ T cells, CD8+ T cells, and natural killer (NK) cells, as well as CD4+ T cell subsets, such as regulatory T cells (Treg), T-helper 1 cells (Th1), T-helper 2 cells (Th2), and T-helper 17 cells (Th17). Additionally, serum cytokine levels, namely Interleukin-2 (IL-2), Interleukin-4 (IL-4), Interleukin-6 (IL-6), Interleukin-10 (IL-10), Interleukin-17 (IL-17), Interferon-γ (IFN-γ), and Tumor necrosis factor-α (TNF-α), were quantified using a magnetic bead-based multiplex immunoassay. The patient’s demographic, clinical, and inflammatory parameters are summarized in **Table 1**. This study was approved by the Ethics Committee of the Second Hospital of Shanxi Medical University (approval no. [2019] YX No. 148 [105]).

**Table 1:** Clinical characteristics of each group.

(A)	Psoriatic Arthritis (a) (*n*= 78)	Seronegative Rheumatoid Arthritis (b) (*n*= 41)	Seropositive Rheumatoid Arthritis (c) (*n*= 79)
Demographics			
Age (years)	46.1 ± 13.19	53.22 ± 11.49 a**	56.18 ± 12.05 a***
Male n (%)	36 (46%)	13 (32%)	20 (25%) a**
Course of disease (month)	14.00 (6.00 - 81.25)	36.00 (9.60 - 72.00) a*	60.00 (24.00 - 192.00) a***
BMI	23.84 ± 4.77	23.23 ± 2.90	23.27 ± 3.42
SJC66	11.91 ± 6.22	–	–
TJC68	7.56 ± 5.84	–	–
SJC28	–	12.02 ± 5.21	8.10 ± 5.07
TJC28	–	11.70 ± 7.03	10.93± 8.47
DAS28 score	–	5.48 ± 1.00	6.07 ± 1.19 b**
Treatment				
NASIDs n (%)	59 (75.64%)	27 (65.83%)	57 (72.15%)
csDMARDs n (%)	41 (52.56%)	23 (46.34%)	47 (59.49%)
bDMARDs n (%)	31 (39.74%)	11 (26.80%)	27 (34.18%)

(B)	Psoriatic Arthritis (a) (*n*= 78)	Seronegative Rheumatoid Arthritis (b) (*n*= 41)	Seropositive Rheumatoid Arthritis (c) (*n*= 79)
Laboratory Characteristics			
ESR, (mm/h)	23.00 (13.00 -51.75)	46.00 (21.00 - 83.00) a**	61.00 (28.50 - 104.00) a***
CRP (mg/L)	10.30 (3.14 -29.55)	13.70 (5.95 - 59.4)	20.00 (7.63 - 65.45) a**
WBC (*10^9^/L)	6.28 (4.88 - 7.87)	5.87 (4.85 - 8.07)	7.44 (5.58 - 8.67)
Hb (g/L)	130.00 (120.00 - 143.50)	118.68 (106.50 - 137.50) a**	114.00 (104.00 - 127.00) a***
PLT (*10^9^/L)	260.50 (213.25 - 308.50)	302.00 (183.50 - 415.50)	362.00 (272.00 - 426.00) a***
LY (*10^9^/L)	1.84 (1.47 - 2.10)	1.45 (1.19 - 1.86) a**	1.54 (1.20 - 2.21) a*
ALT (U/L)	16.65 (11.60 - 23.92)	16.10 (11.10 - 24.80)	13.10 (10.90 - 20.70)
AST (U/L)	19.05 (14.40 - 22.55)	17.80 (13.50 - 24.50)	17.70 (13.30 - 22.50)
BUN (mmol/L)	5.25 (4.12 - 6.27)	5.10 (4.10 - 6.40)	4.60 (3.90 - 6.03)
Cr (umol/L)	54.00 (47.00 - 62.25)	52.00 (45.00 - 61.00)	50.00 (43.00 - 59.90)
CHOL (mmol/L)	3.80 (3.37 - 4.63)	3.83 (3.30 - 4.52)	3.87 (3.27 - 4.68)
TG (mmol/L)	1.32 (0.96 - 1.72)	1.16 (0.88 - 1.44) a*	1.09 (0.76 - 1.39) a**
HDL (mmol/L)	1.07 (0.95 - 1.36)	1.09 (0.89 - 1.39)	1.13 (0.99 - 1.39)
LDL (mmol/L)	2.30 (1.82 - 2.72)	2.27 (1.56 - 2.73)	2.32 (1.98 - 2.93)
IgG (g/L)	12.00 (10.40 - 13.80)	11.60 (9.50 - 14.65)	13.30 (9.61 - 16.70)
IgA (g/L)	2.72 (1.84 - 3.61)	2.69 (1.96 - 3.49)	3.11 (2.34 - 4.48) a*
IgM (g/L)	0.96 (0.72 - 1.30)	0.95 (0.54 - 1.32) c**	1.29 (0.90 - 1.82) a**
RF (U/ml)	–	–	214.17 ± 401.16
Anti-CCP (U/ml)	–	–	803.34 ± 597.08
Anti-MCV (U/ml)	–	–	426.15 ± 390.19

Laboratory Characteristics.

*p < 0.05, **p < 0.01, ***p < 0.001. a, b, c indicate a statistically significant difference compared to the corresponding groups.

BMI, Body mass index; ESR, Erythrocyte sedimentation rate; CRP, C-reactive protein; WBC, White blood cells; Hb, Hemoglobin; PLT, Platelets; LY, Lymphocytes; ALT, Alanine transaminase; AST, Aspartic transaminase; BUN, Blood urea nitrogen; Cr, Creatinine; TG, Triglycerides; CHOL, Cholesterol; LDL, Low density lipoprotein; HDL, High density lipoprotein; IgG, Immunoglobulin G; IgA, Immunoglobulin A; IgM, Immunoglobulin M; RF, Rheumatoid Factor; Anti-CCP, Anti-cyclic Citrullinated Peptide Antibody, Anti-MCV, Anti-mutated Citrullinated Vimentin.

### Statistical analysis

The count data were analyzed using the chi-square goodness-of-fit test. Normality and homogeneity of variance for measurement data were assessed using Kolmogorov-Smirnov and Levene’s tests, respectively, and presented as mean ± standard deviation. Independent sample t-tests were used for two-group comparisons, while the analysis of variance method was used for comparisons among groups. Non-normally distributed data were presented as median (M) and interquartile range and analyzed using the Kruskal-Wallis H test. Using Analysis of Covariance (ANCOVA) to examine and adjust for the impact of covariates on between-group differences. Correlation analysis was conducted using Spearman’s correlation. Statistical significance was defined as p ≤ 0.05. All statistical analyses were performed using SPSS software (version 23.0; SPSS Inc., Chicago, IL, USA).

Univariate binary logistic regression identified risk factors for SpRA, which were integrated into a random forest model to establish an importance ranking based on the mean decrease Gini index (MDG). The variable set with the lowest out-of-bag error estimation rate was chosen for multi-factor binary logistic regression using stepwise random forest analysis. Use receiver operating characteristic curves to assess the discriminative ability of the selected variables for SnRA and SpRA. R software (version 4.2.2, USA) was used for all analysis and visualization.

### The number and percentage of peripheral lymphocyte subsets and CD4+ T cell subsets detected by flow cytometry

**Figure 1:**
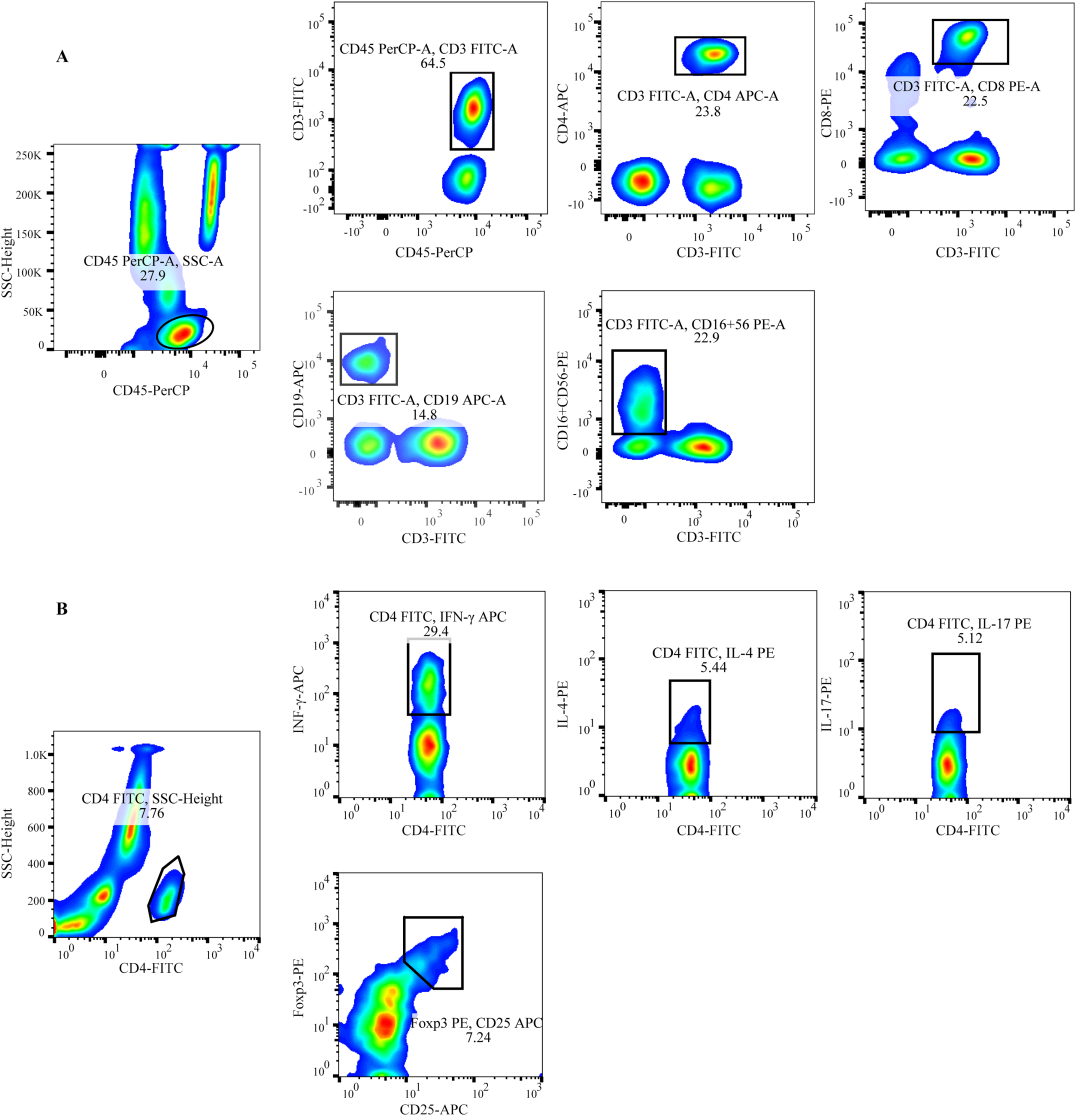
Schematic of the gating for flow cytometric analysis of lymphocyte subsets **(A)** Representative flow cytometry analysis of peripheral lymphocytes.T: CD45+CD3+; CD4+T: CD45+CD3+CD4+; CD8+T: CD45+CD3+CD8+; B: CD45+CD3-CD19+; NK: CD45+CD3-CD16+CD56+ **(B)** Representative flow cytometry analysis of CD4+ T cell subsets. Th1: CD4+INF-γ+; Th2: CD4+IL-4+; Th17: CD4+IL-17+; Treg: CD4+CD25+Foxp3+.

Peripheral blood lymphocytes (T/B/NK/CD4+ T/CD8+T cells) were phenotyped as follows: 50 μL of anticoagulated blood was added to both Trucount A and B tubes. To the A tube, 20 μL of CD3-FITC/CD8-PE/CD45-PerCP/CD4-APC was added, while the B tube received 20 μL of CD3-FITC/CD16+CD56-PE/CD45-PerCP/CD19-APC. Both tubes were incubated for 20 minutes in the dark. Subsequently, 450 μL of hemolysin was added to each tube with gentle mixing. After a 15-minute incubation at room temperature, flow cytometry analysis was performed (**Figure 1A**).

The staining protocol for CD4+T cell subsets included detecting Th1 cells with anti-CD4-FITC/IFN-γ-APC, Th2 cells with anti-CD4-FITC/IL-4-PE, Th17 cells with anti-CD4-FITC/IL-17-PE, and Treg cells with anti-CD4-FITC/CD25-APC/FOXP3-PE. To detect CD4+ T cell subsets (Th1, Th2, and Th17), 80 μL of heparinized blood was mixed with 10 μL of PMA, 10 μL of ionomycin, and 1 μL of Golgi Stop, and then incubated for 5 hours at 37°C. The samples were aliquoted into two tubes, each treated with anti-CD4-FITC and incubated for 30 minutes in the dark. A fixation/permeabilization solution was then applied, followed by an additional 30-minute incubation in the dark. To one tube, anti-IFN-APC and anti-IL-4-PE were added to identify Th1/Th2 cells. In the other tube, anti-IL-17-PE was used to stain Th17 cells. After a further 30-minute incubation in the dark, the cells were washed with PBS and detection was carried out (**Figure 1B**).

For Treg cell detection, 80 μL of anticoagulated blood was incubated with anti-CD4-FITC and anti-CD25-APC for 30 minutes in the dark. Following this, 1 mL of fixation/permeabilization solution was added, and the sample was incubated for an additional 30 minutes in the dark. Finally, anti-Foxp3-PE was added to the blood, and the mixture was incubated for 30 minutes in the dark (**Figure 1B**). All immunofluorescence antibodies were purchased from BD Biosciences (Franklin Lakes, NJ, USA), and blood samples were mixed, incubated, and washed according to the manufacturer’s instructions. Detection was performed using FACSCalibur flow cytometry and BD Multitest software (BD Bio-sciences, Franklin Lakes, NJ, USA) within 24 hours.

### Cytokine level detection

The serum cytokine levels, including IL-2, IL-4, IL-6, IL-10, IL-17, TNF-α, and IFN-γ, were quantified using magnetic bead-based multiplex immunoassay. The Th1/Th2/Th17 subgroup detection kit was procured from Jiangxi Saiji Biotech-nology Co., Ltd. The BioPlex 200 automatic cytokine analyzer was utilized for cytokine data collection. In addition, the BioPlex Manager software was used to determine the cytokine’s median fluorescence intensity (MFI) and concentration (pg/mL). The serum samples were stored at -80°C prior to analysis.

## Results

### Comparison of demographic and laboratory parameters among patient groups

This study included 198 patients (69 males, 129 females; mean age 51.6 ± 13.2 years) and a control group of 53 healthy individuals (mean age 47.68 ± 11.80 years, 32% male). Upon comparison, we found that SpRA patients had higher DAS28 scores than SnRA, consistent with previous findings (14). SnRA and SpRA patients showed elevated ESR levels compared to PsA patients, and SpRA patients also demonstrated higher CRP and platelet levels than PsA patients. Furthermore, it is noteworthy that in SpRA patients, not only were IgA levels higher compared to PsA patients but IgM levels were significantly elevated compared to both SnRA and PsA groups (**Table 1**).

### Differences in peripheral blood lymphocyte subsets and CD4+ T cell levels among each group

**Figure 2:**
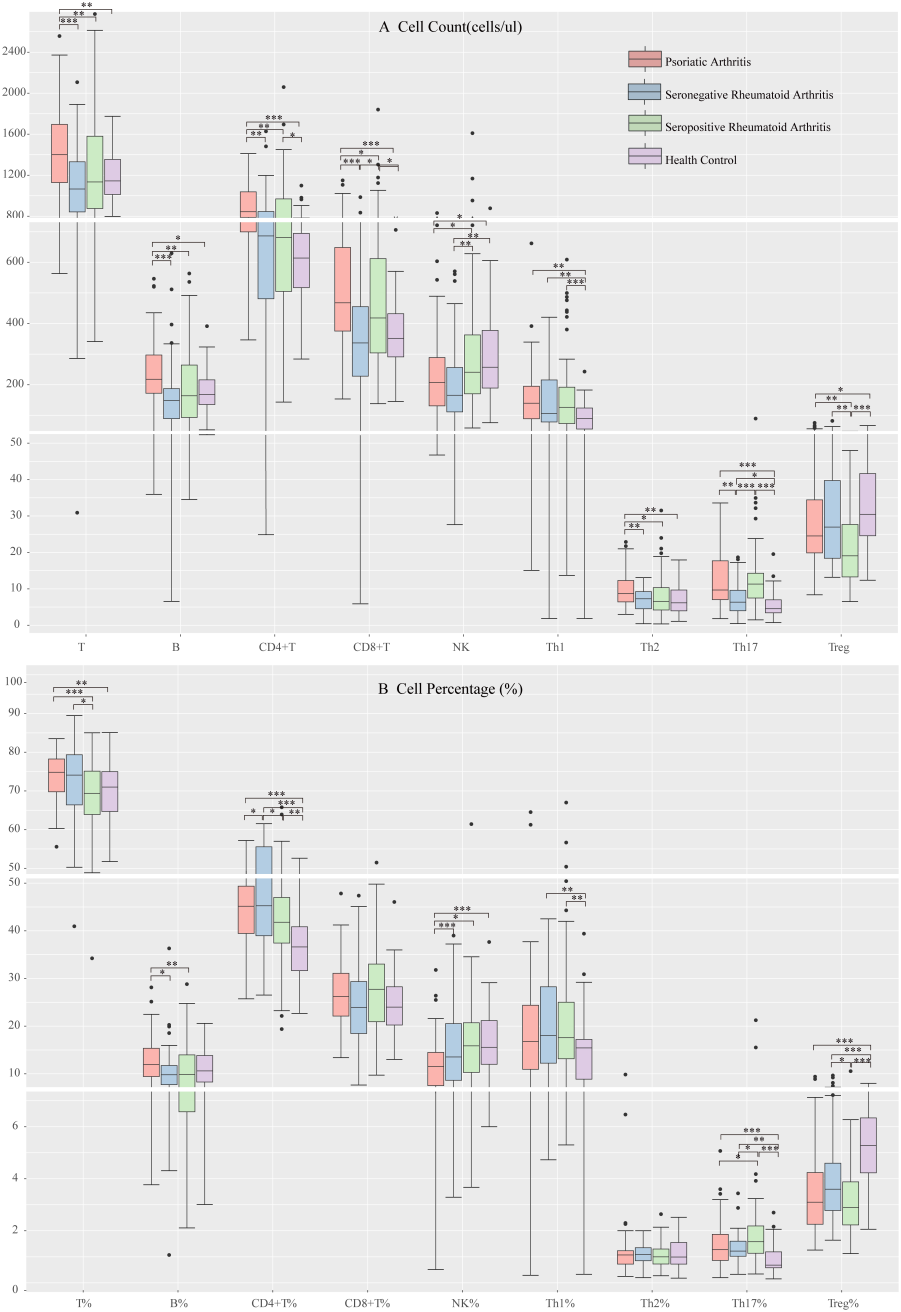
Peripheral blood lymphocyte subpopulation levels in each study group **(A)** Comparison of peripheral blood lymphocyte subsets and CD4^+^ T cell counts among each study group **(B)** Comparison of the proportion of peripheral blood lymphocyte subsets and CD4+ T cells among each study group (*p < 0.05, **p < 0.01, ***p < 0.001; by *ANOVA analysis*).

We compared patient groups’ peripheral blood T, B, and NK cell levels. We found varying degrees of decrease in peripheral blood T, B, and CD4+ T cell counts and B cell percentages in SpRA and SnRA patients compared to those with PsA. Additionally, the rate of T cells was lowest in SpRA, while the number of CD8+ T cells was lowest in SnRA, and the percentage of CD4+ T cells was significantly higher in SnRA compared to the other two groups. Contrary to other lymphocyte subsets, the number and percentage of NK cells were significantly higher in SpRA compared to PsA patients (**Figures 2A, B**).

Next, we compared the levels of CD4+ T cell subsets among the groups. There were no significant differences in Th1 cell levels among the disease groups. PsA patients showed elevated Th2 cell counts compared to the other two groups, although the percentages did not significantly differ. SnRA patients exhibited significantly decreased Th17 cell counts, while in SpRA patients, the reduction was primarily observed in Treg cells (**Figures 2A, B**).

Due to the retrospective cross-sectional nature of this study, baseline differences among enrolled patients were inevitable. Considering that age, gender, disease duration, ESR, and CRP might affect peripheral blood lymphocyte subsets and CD4+ T cell subsets in RA and PsA patients, we utilized ANCOVA to analyze and correct for the influence of these covariates on intergroup differences. After adjusting for the bias introduced by these covariates, significant differences in lymphocyte subsets and CD4+ T cell subsets among the three groups remained (p < 0.05).

Finally, we compared the differences in lymphocyte subsets among PsA, SnRA, SpRA, and healthy individuals. Compared to the patient groups, healthy individuals exhibited significantly lower levels of CD4+ T cells, particularly Th1 and Th17 cells, and significantly higher levels of Treg cells (**Figures 2A, B**).

### SpRA patients exhibit more pronounced Th17/Treg dysregulation

**Figure 3:**
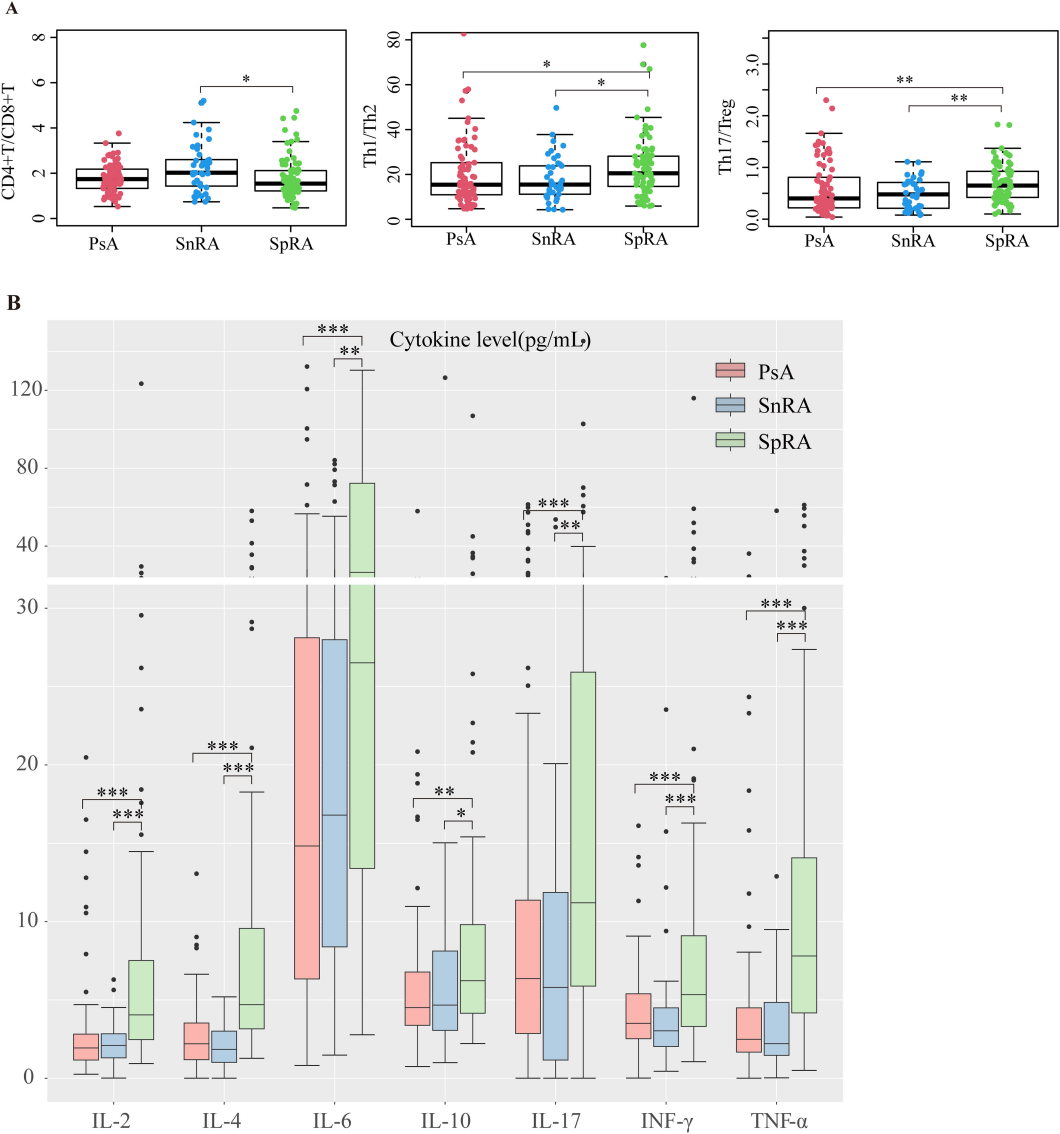
PsA and SnRA display similar peripheral blood immunological profiles **(A)** Differences in CD4+T/CD8+T, Th1/Th2, and Th17/Treg ratios in each study group **(B)** Differences in serum levels of IL-2, IL-4, IL-6, IL-10, IL-17, INF-γ, and TNF-α in each study group (*p < 0.05, **p < 0.01, ***p < 0.001; by *ANOVA analysis*).

Immunological imbalance plays a pivotal role in the pathogenesis of autoimmune diseases. Our study compared CD4+T/CD8+T, Th1/Th2, and Th17/Treg ratios among different groups. SpRA patients exhibited significantly higher Th1/Th2 and Th17/Treg ratios than the other two groups, with the elevation in Th17/Treg being particularly prominent. Additionally, the CD4+T/CD8+T ratio was notably lower in SpRA than in SnRA, while no significant differences were observed between SnRA and PsA patients. These findings underscore a more pronounced immunological imbalance, especially Th17/Treg dysregulation, in SpRA (**Figure 3A**).

### Elevated serum cytokine levels in SpRA

Immunological imbalance precipitates the release of various pro-inflammatory cytokines. Upon comparison of serum cytokine levels across the groups, we observed markedly increased levels of IL-2, IL-4, IL-6, IL-10, IL-17, INF-γ, and TNF-α in SpRA patients compared to the other two groups. Conversely, no significant differences in cytokine levels were noted between SnRA and PsA patients (**Figure 3B**). Similarly, we performed ANCOVA to investigate and correct for the effects of age, gender, disease duration, ESR, and CRP on cytokine levels across the three groups. After adjusting for these covariates, significant differences in cytokine levels among the groups persisted (p < 0.05).

### Similar immunological profiles between SnRA and PsA

Upon analyzing the differences in lymphocyte subsets and cytokine levels, we found that PsA and SnRA exhibited more similar overall lymphocyte distribution patterns compared to SpRA, characterized by comparable CD4+T/CD8+T, Th1/Th2, and Th17/Treg ratios. Additionally, PsA and SnRA shared a similar cytokine profile, with significantly lower serum cytokine levels compared to SpRA patients. This provides immunological evidence for the similar clinical and pathological manifestations between SnRA and PsA (**Figures 3A, B**)

### Stepwise selection of immunological differential factors between SnRA and SpRA

**Figure 4:**
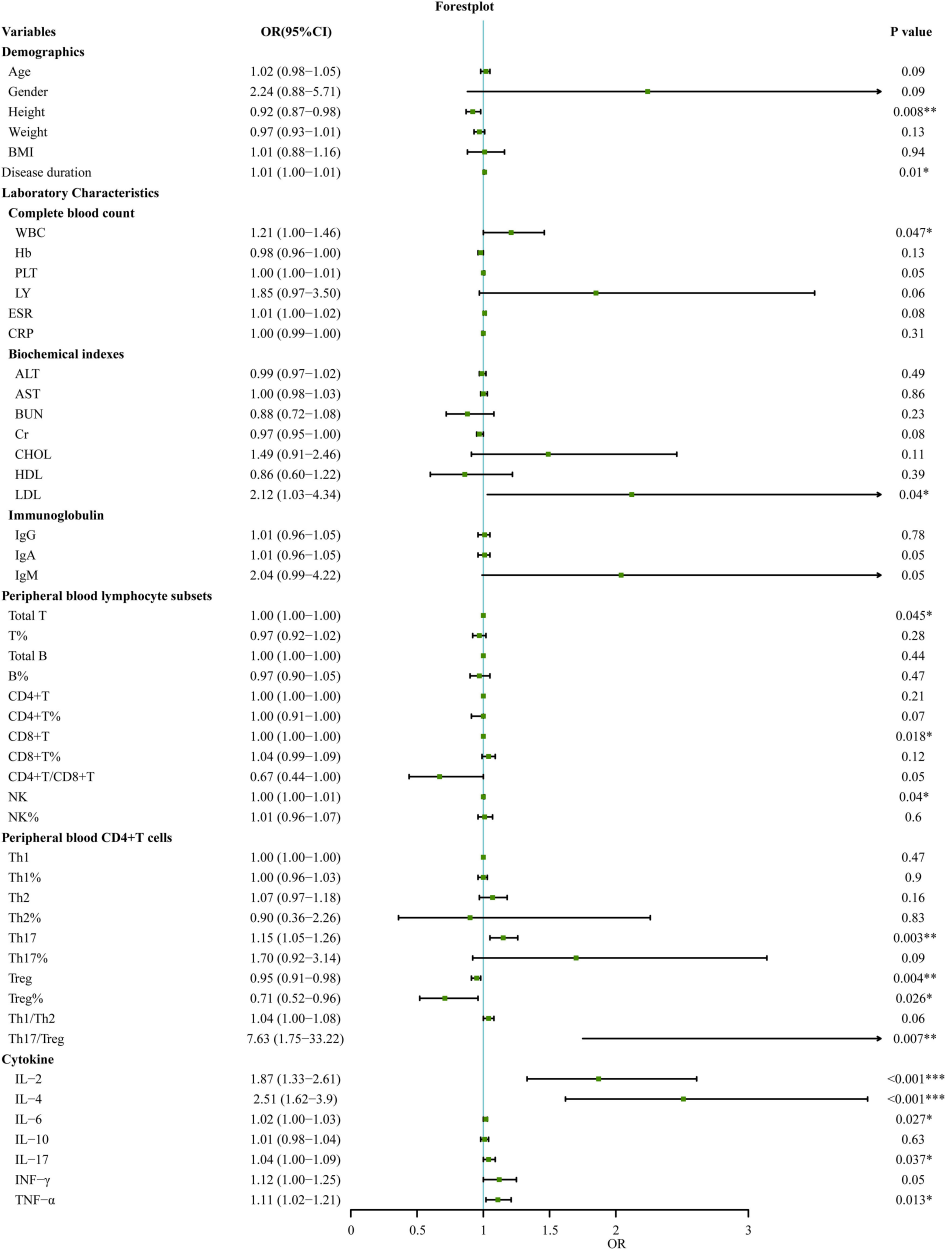
Univariate logistic regression analyses for factors associated with SpRA patients (*p < 0.05, **p < 0.01, ***p < 0.001).

In this study, 120 RA patients were randomly divided into a modeling group (96 cases) and a validation group (24 cases) at an 8:2 ratio using a random number table. None of the measured parameters showed any statistically significant differences between the two groups (p>0.05). Next, we conducted univariate logistic regression analysis on demographic data, clinical indicators, auxiliary examination results, peripheral blood lymphocyte subsets, CD4+T cell subsets, and cytokine levels to identify distinguishing factors between SnRA and SpRA. Univariate logistic regression in the modeling group identified several factors distinguishing SnRA from SpRA patients, including height, disease duration, white blood cell count, LDL, total T cell count, CD8+T cell count, NK cell count, NK cell percentage, Th17 cell count, Treg cell count and percentage, Th17/Treg ratio, IL-2, IL-4, IL-6, IL-17, and TNF-α (p<0.05) (**Figure 4**).

**Figure 5:**
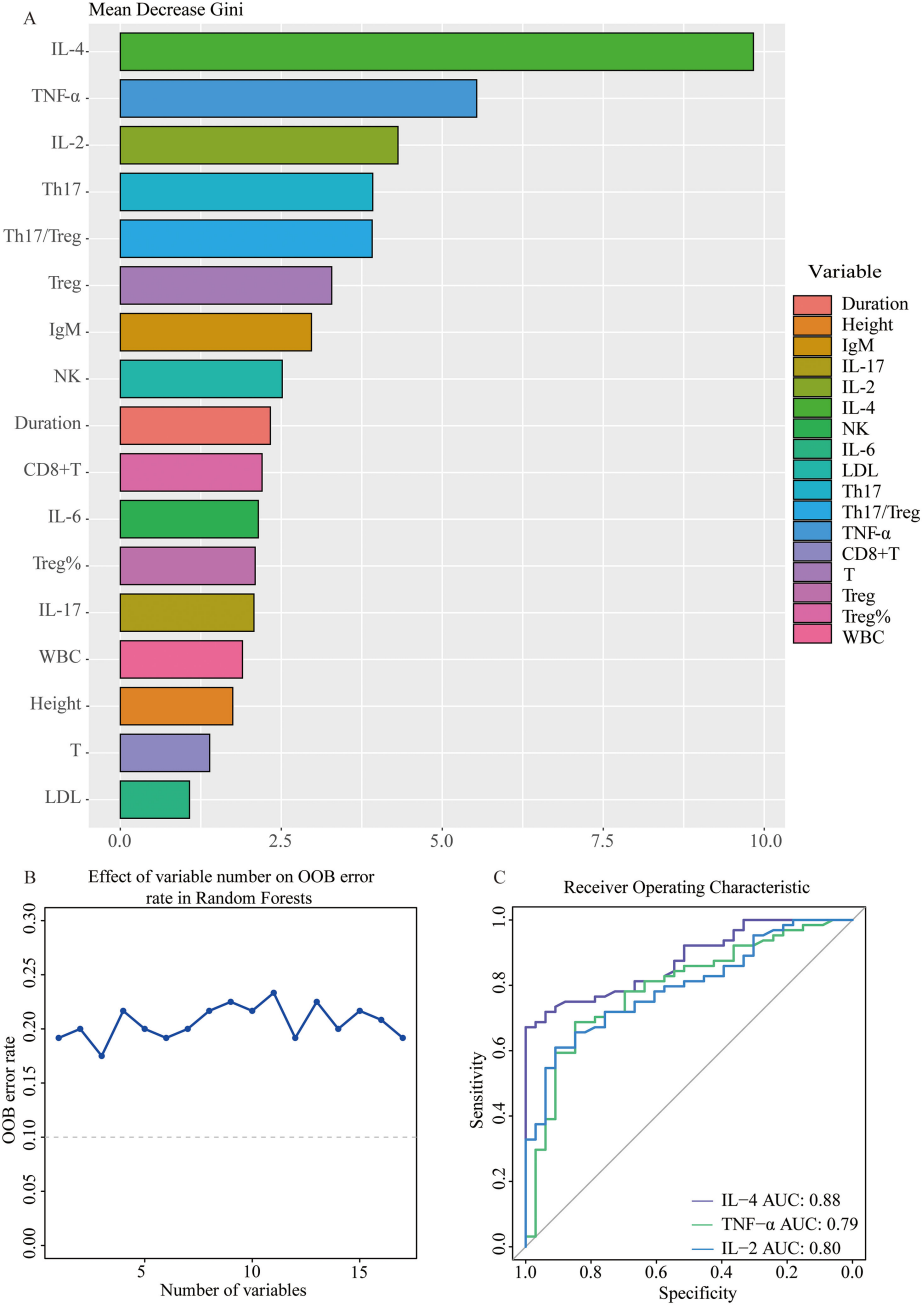
Stepwise selection of key immunological differential factors between SnRA and SpRA **(A)** Variable importance plot using RF model between SnRA and SpRA **(B)** Stepwise Random Forest calculates the out-of-bag estimation error rate corresponding to the number of variables **(C)** Receiver operating curves (ROC) for IL-4, TNF-α, and IL-2.

Next, variables with statistically significant differences identified through univariate logistic regression - height, disease duration, white blood cell count, LDL, total T cell count, CD8+T cell count, NK cell count, NK cell percentage, Th17 cell count, Treg cell count and percentage, Th17/Treg ratio, IL-2, IL-4, IL-6, IL-17, and TNF-α - were included in random forest analysis to explore their importance and predictive value in SnRA and SpRA. Ranking variables based on Mean Decrease Gini (MDG) values in the random forest model (**Figure 5A**), sequential random forest regression analysis was performed from high to low importance. Results showed that reducing the number of variables to 3 minimized the out-of-bag error estimation rate (OBB) (**Figure 5B**). Therefore, the top three variables in MDG ranking, IL-4, TNF-α, and IL-2, were identified as the most critical immunological differences. ROC curves were plotted for these key immunological differences, with AUC values of 0.88 for IL-4, 0.79 for TNF-α, and 0.80 for IL-2, indicating their discriminatory solid ability (**Figure 5C**).

Additionally, it is worth noting that the disparities in Th17 and Treg levels between SpRA and SnRA, alongside the imbalance in the Th17/Treg ratio, emerged as significant immunological indicators in univariate logistic regression (**Figure 4**). These factors ranked 4-6 in terms of variable importance, further confirming the pivotal role of Th17/Treg ratio imbalance in the pathogenesis of both SpRA and SnRA(**Figure 5A**).

### IL-4 level: the key immunological discrepancy between SnRA and SpRA

**Figure 6:**
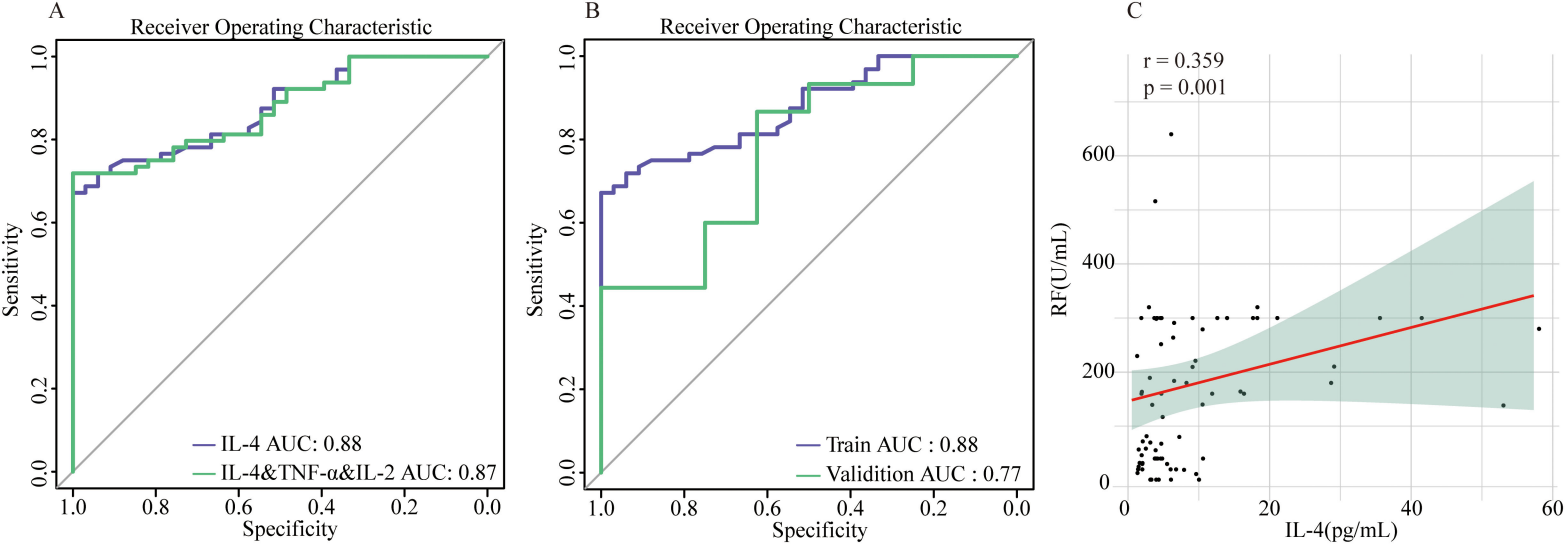
IL-4 as the central immunological difference between SnRA and SpRA **(A)** ROC curves for IL-4 alone and IL-4 in combination with TNF-α and IL-2 **(B)** ROC curves for IL-4 in the modeling group and validation group **(C)** Correlation analysis between serum IL-4 and RF in patients with SpRA.

Further stepwise multivariable logistic regression was conducted on those above key immunological disparities IL-4, TNF-α, and IL-2. Remarkably, only IL-4 levels exhibited statistically significant differences (p=0.001). Subsequently, we compared the discriminative ability between the combined use of these factors and IL-4 alone for distinguishing between the two patient groups. Strikingly, IL-4 independently demonstrated superior discriminative performance (AUC=0.88) compared to the combined use of all three factors(AUC=0.87). Therefore, serum IL-4 levels emerge as the central immunological discrepancy between SnRA and SpRA (**Figure 6A**). Next, we validated the discriminative power of the key immunological difference, IL-4, in the validation cohort, yielding an AUC of 0.77, indicating excellent discriminatory capability within the population (**Figure 6B**).

Given that IL-4 and the Th17/Treg ratio are key distinguishing factors between SnRA and SpRA, we conducted correlation analyses between these immune markers and key disease activity indicators (ESR, CRP, DAS28) as well as antibody titers (RF, Anti-CCP, Anti-MCV) in both groups. In SpRA patients, serum IL-4 levels were positively correlated with RF titers (r=0.359, p=0.001), while no significant correlations were observed for other parameters (p > 0.05). This further supports the role of IL-4 levels as an important factor influencing antibody production and phenotype in RA patients (**Figure 6C**).

## Discussion

Rheumatoid arthritis is a chronic, systemic autoimmune disease characterized by autoantibody production and the destruction of cartilage and bone (15). RF and ACPA play direct roles in various pathological processes of RA, correlating with joint damage, complications, and higher mortality rates (16, 17). The unique seronegative phenotype of SpRA, along with its heterogeneous clinical course and treatment response (18), particularly its similarity to PsA, poses significant challenges in diagnosis and treatment. Current perspectives suggest that SnRA and SpRA are distinct disease entities with different pathogenesis, risk factors, and cytokine profiles (19). Over the years, efforts have been made to identify the characteristic biomarkers of SnRA to achieve precise diagnosis and treatment. However, most studies have focused on individual lymphocyte subsets or cytokines, lacking comprehensive evaluation of the levels of various immune cells and cytokines and their correlations. This study integrates the analysis of peripheral blood lymphocyte subsets, CD4+ T cell subsets, and cytokine profiles of SnRA and SpRA patients, comparing them with those in PsA, aiming to describe better the immune characteristics of these two different disease subtypes. Furthermore, critical immunological differences between SnRA and SpRA are gradually screened to explore the triggering factors of their different antibody statuses.

The immune dysregulation stemming from an imbalance of immune cells represents one of the mechanisms driving the pathogenesis of RA. There is a dearth of studies conducting a systematic comparison of immune cell subpopulations between SpRA and SnRA. Our study revealed that although not statistically significant, SpRA exhibited higher counts of total B cells, T cells, and CD4+ T cells than SnRA, while SpRA showed an elevated number of CD8+ T cells. Importantly, our study also unveiled a noteworthy elevation in NK cell numbers among SpRA patients. NK cells play a distinct role in mediating local inflammation and bone destruction in RA (20). There have been reports on the levels of NK cells with different antibody subtypes of RA. A study by Paulina et al. found that, compared to SnRA, SpRA patients exhibited a decrease in both the number of peripheral blood NK cells and their secretion of INF-γ (21). However, our study reached conclusions opposite to theirs, which may be attributed to geographical differences and variances in flow cytometry gating strategies.

Changes in the levels and functionality of CD4+ T cells, particularly the imbalance between Th17 and Treg cells, play a crucial role in the onset and progression of RA. Through comparative analysis of peripheral blood CD4+ T cell levels in patients with SnRA and SpRA, we have identified that the primary difference in CD4+ T cell levels between these two disease subtypes lies in the imbalance of Th17/Treg cells. Specifically, SpRA patients exhibit elevated Th17 levels, decreased Treg cells, and a more severe imbalance of Th17/Treg cells. Treg cells, crucial for maintaining immune tolerance, are reduced in RA (22) and are not only associated with disease severity but also closely correlated with ACPA’s presence and antibody titers. Additionally, serum levels of Treg cells are significantly lower in ACPA-positive RA patients compared to ACPA-negative RA patients (23), while pro-inflammatory Th17 cells are elevated in ACPA-positive RA (24). Our study further confirms this perspective, highlighting a more pronounced Th17/Treg imbalance in SpRA. Additionally, through univariate logistic regression and random forest analysis, we have identified Th17/Treg imbalance as one of the vital immunological distinctions between SnRA and SpRA.

Cytokines play crucial roles as mediators in immune responses and inflammatory dysfunctions (25). SpRA and SnRA, being distinct disease entities, exhibit differing cytokine profiles. In SpRA, elevated levels of IL-1β, IL-2, IL-1RA, IL-17, IL-4, IL-15, IL-2R, IL-5, MCP-1, MIP-1α, IFN-α, and IL-13 are observed, while SnRA shows a significant increase in IL-10 levels. Our study similarly confirms elevated levels of IL-2, IL-4, IL-17, INF-γ, and TNF-α in the serum of SpRA patients. Additionally, we found a significant increase in IL-6 levels, but IL-10 levels were concurrently elevated in SpRA patients in our study. Further correlation analysis found that, except for IL-17, other cytokines showed varying degrees of correlation with various lymphocyte subsets.

PsA is a chronic, recurring inflammatory joint disease, and differentiating it from SnRA is challenging due to similar antibody phenotypes and clinical symptoms (26). While previous studies have primarily focused on immune phenotype disparities between PsA and SnRA synovial tissues (27, 28), recent research has indicated analogous leukocyte pool compositions in blood specimens from both conditions (29). In our study, we further compared their cytokine profiles, apart from analyzing variations in peripheral blood immune cell phenotypes. We observed no significant differences in primary immune cell ratios like CD4+T/CD8+T, Th1/Th2, and Th17/Treg between the two groups, with cytokine levels exhibiting similarity. However, these indicators showed varying degrees of contrast compared to SpRA, providing an immunological basis for the resemblance in disease features.

Reliable biological markers could be vital to understanding the pathogenic differences between SpRA and SnRA. To identify crucial differentiating factors between SnRA and SpRA, we initially conducted univariate logistic regression analyses on demographic characteristics, laboratory parameters, and immunological markers between the two subtypes. Subsequently, we subjected the differential indicators to random forest analysis to rank their importance. Our findings highlighted serum levels of IL-4, TNF-α, and IL-2 as the most pivotal immunological disparities between the two conditions. Further multivariate logistic regression analysis identified IL-4 as the core immunological differentiator between SnRA and SpRA. Notably, IL-4 alone demonstrated superior discriminatory power compared to the combined IL-4, TNF-α, and IL-2, exhibiting promising diagnostic efficacy in the validation cohort. This suggests that IL-4 may significantly trigger the distinct antibody phenotypes observed between the two subtypes.

IL-4, an essential member of the Th2 cytokine family, not only effectively stimulates B-cell proliferation but also promotes the conversion of immunoglobulins to IgE and IgG4, playing a crucial role in allergic reactions and parasitic infections (30). Multiple studies have consistently highlighted the role of IL-4 in autoantibody synthesis in RA. On the one hand, IL-4 gene polymorphisms, particularly the rs2243250 variant, are prevalent in RA and are associated not only with disease severity parameters such as ESR, CRP, and DAS28 (31), but also with erosive RA and the formation of anti-CCP antibodies (32). On the other hand, IL-4 deficiency reduces the titers of pathogenic autoantibodies, slowing down the progression of arthritis (33). Recent research has suggested that IL-4 receptor blockade may increase the risk of acute serum-negative arthritis and psoriasis (34, 35). This effect could be attributed to the diminished Th2 immune response and compensatory activation of the IL-23/IL-17 axis following IL-4/IL-13 signaling blockade (34). Our study demonstrates that IL-4 is the key immunological difference between SnRA and SpRA, with serum IL-4 levels showing a significant positive correlation with RF titers in SpRA. Hence, the varying levels of IL-4 involvement in autoantibody synthesis in RA may be crucial for the antibody phenotype disparities observed between SnRA and SpRA.

Indeed, our study has certain limitations. Firstly, the data used for modeling were obtained from a single medical center, lacking external validation. Secondly, as this study is retrospective in nature, it did not adequately account for the effects of various drugs and treatment regimens on patients’ lymphocyte subsets and cytokine profiles. The final and most important point, further investigation is warranted to explore potential disparities in IL-4 gene polymorphisms between SnRA and SpRA and the molecular mechanisms underlying IL-4-induced production of distinct antibody spectra in RA. Additionally, given the similar clinical features and immunophenotypes between PsA and SnRA, investigating whether their treatment strategies and outcomes are comparable, and how they differ from SpRA, particularly with the use of IL-17, IL-4, and TNF-α inhibitors, could be a fascinating and promising area of research.

## Conclusion

This retrospective cross-sectional study comprehensively analyzes differences in peripheral blood lymphocyte subsets and cytokine profiles among SnRA, SpRA, and PsA patients. It reveals that SpRA exhibits more pronounced Th17/Treg dysregulation, highlighting it as one of the vital immunological differences between SpRA and SnRA. In contrast, the distribution characteristics of immune cells and cytokines in SnRA resemble those in PsA patients, providing immunological evidence for the clinical and pathological similarities. Furthermore, IL-4 emerges as the most crucial immunological difference between SnRA and SpRA and may be a critical triggering factor for the distinct antibody phenotypes between SnRA and SpRA.

## Data Availability

The original contributions presented in the study are included in the article/supplementary material. Further inquiries can be directed to the corresponding author.
